# Triglyceride glucose index and its combination with the Get with the Guidelines-Heart Failure score in predicting the prognosis in patients with heart failure

**DOI:** 10.3389/fnut.2022.950338

**Published:** 2022-09-08

**Authors:** Su Han, Chuanhe Wang, Fei Tong, Ying Li, Zhichao Li, Zhaoqing Sun, Zhijun Sun

**Affiliations:** Department of Cardiovascular Medicine, Shengjing Hospital of China Medical University, Shenyang, Liaoning, China

**Keywords:** heart failure, triglyceride glucose index, type 2 diabetes, prognosis, GWTG-HF risk score

## Abstract

**Background:**

Heart failure (HF) is associated with generalized insulin resistance (IR). Recent studies demonstrated that triglyceride glucose (TyG) is an effective alternative index of IR. However, the relationship between the TyG index and in-hospital mortality in patients with HF is unclear. In the present study, we aimed to clarify the association between the TyG index and in-hospital mortality in patients with HF.

**Methods:**

A retrospective study consisting of 4,411 patients diagnosed with HF from 2015 to 2018 was conducted. All-cause mortality during hospitalization was the primary endpoint. The association between the TyG index and in-hospital mortality was assessed using the logistic regression analysis.

**Results:**

The risk of in-hospital mortality was significantly associated with increased TyG index (OR: 1.886, 95% CI: 1.421–2.501, *p* < 0.001) under logistic regression with multivariable adjustment. When divided into three groups based on the TyG index, Tertile 3 demonstrated significantly higher in-hospital mortality than the other two Tertiles (OR: 2.076, 95% CI: 1.284–3.354, *p* = 0.001). Moreover, the TyG index improved the prediction efficiency of the Get with the Guidelines-Heart Failure (GWTG-HF) score (absolute integrated discrimination improvement = 0.006, *p* < 0.001; category-free net reclassification improvement = 0.075, *p* = 0.005). In subgroup analysis, the TyG index exhibited similar predictive performance of in-hospital mortality when groups were stratified based on type 2 diabetes mellitus (T2DM) and coronary artery disease (CAD).

**Conclusion:**

TyG is a potential index for predicting in-hospital mortality in patients with HF, independent of T2DM or CAD status. The TyG index may be combined with the GWTG-HF score to further improve its predictive efficacy.

## Introduction

Heart failure (HF) is the end stage of many cardiovascular diseases, with high morbidity and mortality (1, 2). Due to the continuous development of oral drugs, the poor prognosis of HF has improved to some extent in recent years. However, poor prognosis remains the main cause of mortality in cardiovascular disease (3). Therefore, early risk stratification and accurate identification of high-risk patients with HF are essential for their effective management and treatment.

Insulin resistance (IR) is common in cardiovascular disease and has been established as a predictor of outcomes independent of type 2 diabetes mellitus (T2DM) status (4–7). As research has progressed, IR has been found to play a crucial role in the development of HF because of associated reductions in cardiac insulin metabolic signaling (8–10). To date, the closest approach to the gold diagnostic standard in IR is hyperinsulinemic–euglycemic clamp (11). However, the complexity of the test process limits its clinical applications. Based on the features of IR, hyperglycemia, and dyslipidemia, the triglyceride glucose (TyG) index was developed. TyG is calculated using fasting plasma glucose (FPG) and triglycerides, which are easily obtained by blood tests (12, 13). Numerous clinical studies indicated that TyG is significantly associated with IR obtained by the hyperinsulinemic–euglycemic clamp and even shows a better performance than the homeostasis model assessment of IR (HOMA-IR) in patients with and without diabetes (12, 13).

Previous studies primarily focused on the correlation between the TyG index and coronary artery disease (CAD; 6, 7). However, no study analyzed the predictive value of TyG for adverse events in HF patients with or without T2DM. Therefore, in this study, we aimed to explore the relationship between TyG levels and in-hospital mortality in patients with HF. Furthermore, we evaluated whether TyG could enhance the prediction efficiency of the Get with the Guidelines-Heart Failure (GWTG-HF) score.

## Materials and methods

### Study population and design

The current study population was based on a retrospective observational cohort study, which enrolled 5,126 consecutive patients with HF at the Shengjing Hospital of China Medical University (from January 2015 to December 2018). The diagnosis of HF was confirmed by symptoms (such as breathlessness, ankle swelling, and fatigue), signs (such as elevated jugular venous pressure, pulmonary crackles, and peripheral edema), echocardiography, and the results of laboratory tests as recommended by the modified Framingham criteria (14), including *de novo* HF or decompensation of chronic HF. HF with reduced ejection fraction was defined as left ventricular ejection fraction (LVEF) ≤ 40%, HF with mildly reduced ejection fraction as 41% ≤ LVEF ≤ 49%, and HF with preserved ejection fraction (HFpEF) as LVEF ≥ 50% (15). Patient data were recorded in a database set up specifically for this study. The TyG index was calculated according to the formula:


$$T\,y\,G=I\,n\,\left[f\,a\,s\,t\,i\,n\,g\,t\,r\,i\,g\,l\,y\,c\,e\,r\,i\,d\,e\,s\,\left(\frac{m\,g}{d\,L}\right)\,\;\times f\,a\,s\,t\,i\,n\,g\,p\,l\,a\,s\,m\,a\,g\,l\,u\,c\,o\,s\,e\,\left(\frac{m\,g}{d\,L}\right)÷2\right]$$

The GWTG-HF risk score was calculated based on seven previously reported indicators: systolic blood pressure, heart rate, age, sodium, blood urea nitrogen (BUN), history of chronic obstructive pulmonary disease, and race (16). Within 24 h of admission, fasting venous blood samples were collected from all the patients. FPG and triglyceride levels were measured by an enhanced immunonephelometric assay using an automated analyzer (AU5800; Beckman Coulter, Inc., Carlsbad, CA, United States) in the Shengjing Hospital core laboratory. All-cause in-hospital mortality was the primary endpoint.

The exclusion criteria were as follows: no availability of fasting triglyceride or FPG data, cardiogenic shock, chronic kidney failure with dialysis on admission and/or diagnosed liver disease, and hormone therapy before admission. The present study included 4411 patients with HF.

**FIGURE 1:**
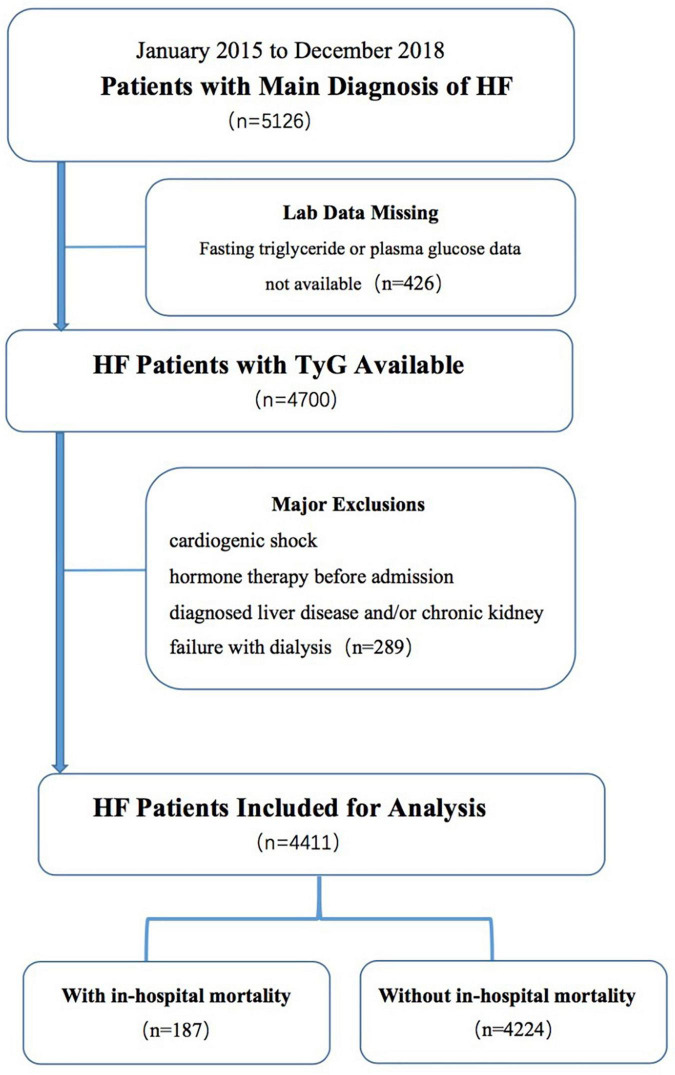
Flowchart of participant selection.

Patient selection is shown in the flow diagram in Figure 1. The participants were divided into two groups according to their in-hospital mortality. This study complied with the Helsinki Declaration and was approved by the Research Ethics Committee of Shengjing Hospital of China Medical University (IRB number: 2019PS594K).

### Statistical analysis

All patients were divided into two groups according to in-hospital mortality. Continuous variables are expressed as mean ± standard deviation, or median and interquartile range, depending on whether or not they were normally distributed. The differences in continuous variables between groups were analyzed using Student’s *t*-test or the Kruskal–Wallis *H* test. Categorical variables are expressed as numbers and percentages (%). The differences in categorical variables between the groups were analyzed using the chi-square test or Fisher’s exact test. Predictors associated with the primary endpoint were analyzed using univariate and multivariate logistic regression analyses. TyG levels were considered to be a continuous variable. To further explore the association between TyG and in-hospital mortality, TyG was also analyzed as a categorical variable, with Tertile 1 as the reference group. The relationship between the TyG index and primary endpoint was given as an odds ratio (OR) with associated 95% confidence intervals (CIs). The predictive potential of the TyG index and TyG + GWTG-HF score was analyzed. In brief, the area under the curve associated with the primary endpoint through the receiver operating characteristic curve was calculated by the statistical software MedCalc (version 18.1.1; MedCalc Software, Ostend, Belgium; 17). Absolute integrated discrimination improvement (IDI) and category-free net reclassification improvement (NRI) were used to evaluate the improvement in predictive efficiency of the TyG + GWTG-HF score compared to the GWTG-HF score (18). The interaction was tested with a likelihood ratio test, and the ORs with associated 95% CIs were represented by a forest plot. Statistical analyses were performed using SAS (version 9.4; Statistical Analysis Software Institute, Inc., Cary, NC, United States), and statistical significance was set at a *p*-value < 0.05.

## Results

### General characteristics

A total of 4,411 patients with HF (mean age: 70.6 ± 12.6) were enrolled in our study, and 48.4% of patients were men. The mean length of hospitalization was 10.0 ± 6.2 days, and 187 (4.2%) patients died during hospitalization. The proportions of patients with HF with reduced ejection fraction, HF with mildly reduced ejection fraction, and HFpEF were 18.9, 40.1, and 41%, respectively. The baseline characteristics of the patients are summarized in Table 1. Patients with in-hospital mortality were likely to have a poor New York Heart Association (NYHA) grade, older age, high systolic blood pressure on admission, higher TyG index, higher GWTG-HF score, and a history of CAD. The patients who met in-hospital mortality also had higher creatinine, BUN, uric acid, N-terminal brain natriuretic peptide and cardiac troponin I levels, and lower hemoglobin, serum sodium, and albumin levels on admission than surviving patients. In terms of medications, the in-hospital mortality group had lower treatment rates with angiotensin-converting enzyme inhibitors (ACEI)/angiotensin receptor blockers (ARB)/angiotensin receptor-neprilysin inhibitor (ARNI), diuretics, and aldosterone antagonists (Table 1).

**TABLE 1:** Characteristics of subjects divided by in-hospital mortality, median (IQR), or N (%), or means ± SD.

Variable	Overall (*n* = 4,411)	Patients with in-hospital mortality (*n* = 187)	Patients without in-hospital mortality (*n* = 4,224)	*P*-value
Age (years)	70.6 ± 12.6	77.1 ± 10.4	70.3 ± 12.6	<0.001
Men [*n* (%)]	2,927 (48.4)	83 (44.4)	2,205 (52.2)	0.036
**NYHA grading [*n* (%)]**				<0.001
II	897 (20.3)	6 (3.2)	891 (21.1)	
III	1,712 (38.8)	40 (21.4)	1,672 (39.6)	
IV	1,802 (40.9)	141 (75.4)	1,661 (39.3)	
Heart rate on admission, bpm	88.2 ± 22.4	89.1 ± 21.1	88.1 ± 22.5	0.552
SBP on admission, mmHg	137.3 ± 23.4	132.5 ± 27.2	137.5 ± 23.2	0.004
GWTG-HF score	57.2 ± 8.8	63.3 ± 7.5	57.9 ± 8.7	<0.001
TyG index	8.58 ± 0.68	8.69 ± 0.74	8.57 ± 0.68	0.024
Triglyceride, mg/dl	111.4 ± 84.2	109.1 ± 75.6	111.6 ± 84.5	0.701
FPG, mg/dl	118.2 ± 44.0	138.3 ± 66.8	117.3 ± 44.7	<0.001
Albumin, g/L	37.1 ± 4.3	34.3 ± 4.6	37.2 ± 4.3	<0.001
TBIL, μmol/L	16.2 ± 11.2	17.4 ± 13.3	16.2 ± 11.1	0.145
LDL, mmol/L	2.58 ± 0.97	2.53 ± 1.06	2.58 ± 0.96	0.438
BUN, mmol/L	8.7 ± 5.3	15.5 ± 9.7	8.5 ± 4.8	<0.001
Creatinine, mg/dl	1.15 ± 0.87	1.84 ± 1.29	1.12 ± 0.83	<0.001
eGFR, ml/min/1.73 m^2^	74.6 ± 30.1	49.4 ± 29.3	75.7 ± 29.7	<0.001
Uric Acid, μmol/L	444.1 ± 144.5	539.0 ± 216.7	436.8 ± 138.9	<0.001
Hemoglobin, g/L	126.6 ± 23.0	112.4 ± 28.5	127.2 ± 22.5	<0.001
HbA1c, %	6.47 ± 1.36	6.61 ± 1.47	6.46 ± 1.35	0.156
Serum sodium, mmol/L	139.1 ± 3.9	137.4 ± 5.9	139.2 ± 3.8	<0.001
cTNI, ng/ml	0.04 (0.01, 0.21)	0.19 (0.05, 2.6)	0.04 (0.01, 0.19)	<0.001
NT-proBNP, pg/ml	5,036 (1,592, 5,328)	5,327 (4,978, 11,833)	4,718 (1,494, 5,329)	<0.001
LVEF, %	49.1 ± 10.4	47.9 ± 8.2	49.2 ± 10.5	0.105
**Comorbidities, *n (%)***				
CAD	3,133 (71.0)	147 (78.6)	2,986 (70.7)	0.020
Hypertension	2,778 (63.0)	109 (58.3)	2,669 (63.2)	0.175
AF	1,375 (31.2)	43 (23.0)	1,332 (31.5)	0.014
T2DM	1,414 (32.1)	70 (37.4)	1,344 (31.8)	0.107
COPD	1,051 (23.8)	37 (19.8)	1,014 (24.0)	0.185
Smoking, *n (%)*	1,222 (27.7)	35 (18.7)	1,187 (28.1)	0.005
**Medications, *n (%)***				
ACE-I/ARB/ARNI	3,581 (81.2)	113 (60.4)	3,468 (82.1)	<0.001
Beta blockers	3,592 (81.4)	159 (85.0)	3,433 (81.3)	0.196
Diuretic	3,724 (84.4)	141 (75.4)	3,583 (84.8)	0.001
Aldosterone antagonists	1,284 (29.1)	41 (21.9)	1,243 (29.4)	0.027

ACEI, angiotensin-converting enzyme inhibitors; AF, atrial fibrillation; ARB, angiotensin II receptor blockers; ARNI, angiotensin receptor blocker-neprilysin inhibitors; BUN, blood urea nitrogen; CAD, coronary artery disease; COPD, chronic obstructive pulmonary disease; cTNI, cardiac troponin I; eGFR, estimated glomerular filtration rate; FPG, fasting plasma glucose; GWTG-HF, Get With the Guidelines-Heart Failure; HbA1c, glycated hemoglobin; LDL, low-density lipoprotein; LVEF, left ventricular ejection fraction; NT-proBNP, N-terminal brain natriuretic peptide; SBP, systolic blood pressure; T2DM, type 2 diabetes mellitus; TBIL, total bilirubin; TyG, triglyceride glucose.

### Ability of triglyceride glucose index to predict prognosis

Indicators of the primary endpoint identified by univariate analysis were also analyzed using multivariate analysis. Supplementary File 1 shows the univariate logistic analysis and the indicators related to in-hospital mortality: TyG index, age, sex, NYHA grade, systolic blood pressure, albumin, total bilirubin, BUN, creatinine, uric acid, hemoglobin, serum sodium, N-terminal brain natriuretic peptide, cardiac troponin I, LVEF, history of CAD, atrial fibrillation, smoking, and the use of ACEI/ARB/ARNI, beta-blockers, diuretics, or aldosterone antagonists.

Moreover, the logistic univariate analysis demonstrated that TyG, as a continuous variable, was associated with in-hospital mortality (OR: 1.266, 95% CI: 1.031–1.551, *p* = 0.024). Multiple adjustments for confounders did not attenuate this prediction (OR: 1.886, 95% CI: 1.421–2.501, *p* < 0.001) (Table 2). When patients were divided into three groups based on their TyG index, TyG remained a strong predictor of the primary endpoint. Using Tertile 1 (TyG index < 8.25) as a reference, multivariate analysis revealed that the TyG index in Tertiles 2 (8.25 ≤ TyG index < 8.78) and 3 (TyG index ≥ 8.78) increased the OR for in-hospital mortality in patients with HF (Tertile 2: OR 1.536, 95% CI 1.002–2.354, *p* = 0.049; Tertile 3: OR 2.076, 95% CI 1.284–3.354, *p* = 0.003) (Table 2) (The details of the multivariate analysis are shown in Supplementary Files 2, 3).

**TABLE 2:** Effects of multiple variables on clinical outcomes in univariate and multivariate analyses.

	*Univariate analysis*			*Multivariate analysis*		
		
	*OR*	*95% CI*	*P*	*OR*	*95% CI*	*P*
GWTG-HF, per 1 score increase	1.099	1.078–1.121	<0.001	–	–	–
**TyG as a continuous variable**						
TyG, per 1 score increase	1.266	1.031–1.554	0.024	1.886	1.421–2.501	<0.001*
**TyG as a categories variable**						
Tertile 1	Reference			Reference		
Tertile 2	1.054	0.726–1.531	0.781	1.536	1.002–2.354	0.049*
Tertile 3	1.301	0.911–1.860	0.148	2.076	1.284–3.354	0.003*

*Adjusted for age, sex, NYHA grading, heart rate on admission, SBP on admission, albumin, TBIL, LDL, BUN, creatinine, uric acid, hemoglobin, serum sodium, cTNI, NT-proBNP, LVEF, and the history of CAD, hypertension, AF, DM, COPD, smoking, ACEI/ARB/ARNI, beta-blockers, diuretic, aldosterone antagonists.

### Prognostic accuracy of triglyceride glucose and comparison of different parameters

Combining the TyG index and GWTG-HF score enhanced the predictive efficiency of the new model compared to that with the GWTG-HF score only (IDI = 0.006, *p* < 0.001; NRI = 0.075, *p* = 0.005) (Table 3).

**TABLE 3:** Comparisons of the predictive performance of GWTG-HF and TYG+GWTG-HF for the prognosis prediction.

	AUC (95%CI)	z for C-statistic	P for C-statistic	NRI	P for NRI	IDI	P for IDI
GWTG-HF score	0.712 (0.698–0.725)	–	–	–	–	–	–
TyG+GWTG-HF vs. GWTG-HF	0.720 (0.706–0.733)	1.195	0.232	0.075	0.005	0.006	<0.001

AUC, area under the curve; GWTG-HF, Get with the Guidelines-Heart Failure score; TyG, triglyceride glucose.

**FIGURE 2:**
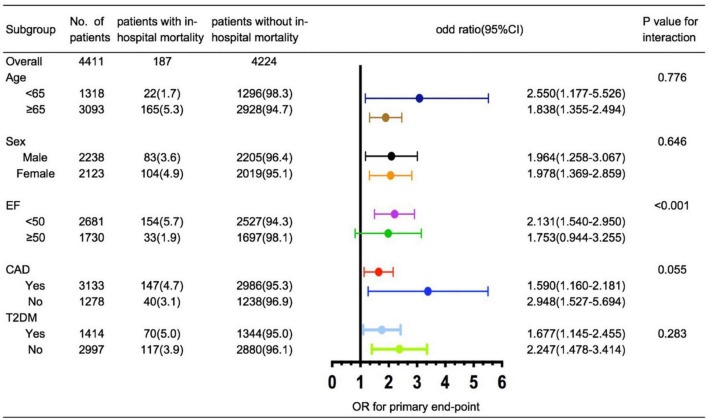
*Post hoc* subgroup analysis of TyG index for the primary endpoint.

Relevant clinical indicators for in-hospital mortality such as age (< 65 vs. ≥ 65 years), ejection fraction (< 50% vs. ≥ 50%), sex (men vs. women), CAD (yes vs. no), and T2DM (yes vs. no) were evaluated using *post hoc* subgroup analysis. In subgroup analysis, we observed that a higher TyG index was significantly associated with an increased risk of in-hospital mortality after stratification by age, sex, CAD, and T2DM. However, the TyG index was less effective for predicting the endpoint in patients with HFpEF (Figure 2).

## Discussion

The TyG level was found to be a potential independent index for predicting in-hospital mortality. Furthermore, when combined with the GWTG-HF score, the TyG index produced better prognostic efficiency than the GWTG-HF score alone. Additionally, a similar predictive performance was found when patients were stratified by CAD and T2DM, which indicates that the TyG index plays an important role in prediction, independent of T2DM or CAD status. Therefore, our study reveals a relationship between TyG and HF in the general population for the first time.

Insulin resistance is classically defined as the failure of insulin to promote its metabolic activity in organs and tissues, such as the liver, the skeletal muscle, and the adipose tissue (8, 9). In 2005, Ingelsson et al. reported that IR could predict HF incidence even when adjusted for established risk factors for HF (19). In their study, insulin sensitivity was measured using the euglycemic insulin clamp (20). Despite the improvements in accuracy from euglycemic insulin and hyperinsulinemic–euglycemic clamps, these complex procedures remain difficult to use in the clinic. In the ARIC study, Vardeny et al. utilized HOMA-IR, which was calculated using FPG and fasting insulin, to explore the relationship between IR and HF. They observed that lower levels of HOMA-IR (1.0–2.0) than previously considered were associated with an increased risk of HF (21). Other than FPG and insulin, the symptoms of IR may manifest as dyslipidemia due to its influence on the liver (5, 22). Based on glucotoxicity and lipotoxicity, TyG was developed as a potential index to represent IR. In previous studies, TyG has shown a good correlation with IR, better than HOMA-IR (12, 13). Wenqin et al. studied 546 HF patients with T2DM and demonstrated a positive correlation between the TyG index and prognosis (23). However, the predictive effect of the TyG index in patients with HF who are non-diabetic remains unclear. To the best of our knowledge, our study is the first to explore the prognostic value of the TyG index in patients with HF, independent of T2DM status. We observed that TyG is a potential independent index for predicting in-hospital mortality in patients with HF. Moreover, the TyG index showed good performance in patients who are non-diabetic with HF (interaction *p* = 0.283, Figure 2). This observation suggests that IR affects the prognosis of patients with HF independent of diabetes. To further explore the clinical conditions of patients, we performed a subgroup analysis. A similar predictive performance was found for the TyG index under CAD stratification (interaction *p* = 0.055, Figure 2). This result was supported by the ARIC study, indicating that the relationship between IR and HF is not mediated by an increased risk of CAD (21). Notably, the results of our subgroup analysis of LVEF differ from those of previous studies (24, 25). In a prospective study of HF patients with LVEF > 40%, Tamariz et al. reported that patients with metabolic syndrome had a worse prognosis at 2.6 years of follow-up than those without metabolic syndrome (26). In contrast, Zhou et al. studied 1,548 patients with HFpEF and found no differences in cardiovascular and all-cause mortality between groups with and without metabolic syndrome. This is consistent with the results of our subgroup analyses. The predictive efficiency of the TyG index was impaired in patients with HFpEF (Figure 2). This result may be related to the heterogeneous nature of HFpEF (27). Age, female sex, diabetes, hypertension, obesity, metabolic syndrome, and atrial fibrillation have been identified as classical risk factors for the development of HFpEF (28). Thus, interactions between these comorbidities may make it challenging to evaluate specific indicators of in-hospital mortality. In addition, the 95% CI for the TyG index in HFpEF was wide and included a potentially important effect size; therefore, further understanding of the pathophysiology of HFpEF and long-term follow-up studies may be required to assess the TyG index for patients with HFpEF.

In 2010, Peterson et al. set up the GWTG-HF score to predict adverse events in hospitals, and the points-scoring system has been widely utilized since the publishing of studies by Peterson et al., Suzuki et al., and Shiraishi et al. (16, 29, 30). We further explored whether TyG can enhance the predictive efficiency of the GWTG-HF score. Because the receiver operating characteristic analysis could not provide pertinent information about whether a combination of GWTG-HF score with a new index would enhance the accuracy of the model for predicting the risk of in-hospital mortality, we also conducted IDI and NRI to measure the incremental value (31, 32). In our study, both IDI and NRI demonstrated a significant improvement in the reclassification of in-hospital mortality by TyG + GWTG-HF vs. GWTG-HF alone (IDI = 0.006, *p* < 0.001; NRI = 0.075, *p* = 0.005) (Table 3).

Although the pathophysiology of IR in HF requires further research, the effect of the TyG index on patient prognosis may be related to known mechanisms. In conditions of IR, the myocardium utilizes free fatty acids and decreases glucose uptake and oxidation (33). Because glucose is a more efficient substrate than fatty acids, converting cardiac metabolism from glucose metabolism to fatty acid oxidation reduces cardiac efficiency. This metabolic disorder increases the predisposition to stress overload and ischemia. In addition to glucotoxicity and lipotoxicity, the dysregulation of neurohumoral factors, cytokines, and oxidative stress is the main cause of cardiac IR and impaired cardiac function (34). Furthermore, IR can lead to hyperinsulinemia, which is recognized as a factor that accelerates cardiac remodeling (35, 36). Hyperinsulinemia can also lead to the retention of sodium and the activation of the sympathetic nervous system, which contributes to HF (37, 38). Consequently, IR influences glucose and lipid metabolism, leading to a mismatch in energy needs, affecting neurohumoral factors, and causing HF progression.

The TyG index is simple to calculate and easy to obtain at the bedside. Therefore, it improves upon other IR prediction indicators and must be popularized. Monitoring the TyG index can better identify patients with a high risk of in-hospital mortality from HF, independent of T2DM and CAD status. Additionally, a new model consisting of the TyG index and GWTG-HF score can further enhance the predictive efficacy of GWTG-HF, potentially benefiting clinical practice.

The present study has some limitations. First, this was a retrospective study, and the causal relationship of the association between the TyG index and in-hospital mortality requires further confirmation through prospective studies. Second, because the TyG index was assessed only at admission without dynamic monitoring, we could not assess whether lowering the TyG index might improve patient prognosis. Finally, although we included as many clinically relevant variables as possible in the multivariate analysis, potential confounders likely remained.

In summary, TyG was a potential independent index for predicting in-hospital mortality in patients with HF, independent of T2DM or CAD status. The TyG index can further improve the predictive efficacy of the GWTG-HF score.

## Data availability statement

The raw data supporting the conclusions of this article will be made available by the authors, without undue reservation.

## Ethics statement

Ethical review and approval was not required for the study on human participants in accordance with the local legislation and institutional requirements. Written informed consent from the patients was not required to participate in this study in accordance with the national legislation and the institutional requirements.

## Author contributions

All authors were involved in the conception and design of the study, as well as in the collection, analysis and interpretation of data, reviewed, and approved the final manuscript.
