# Supplementary material for: Triglyceride glucose index and its combination with the Get with the Guidelines-Heart Failure score in predicting the prognosis in patients with heart failure
Source: Front Nutr. 2022 Sep 8;9:950338. doi: 10.3389/fnut.2022.950338 (PMC9493032; doi:10.3389/fnut.2022.950338)
Supplement: Supplementary file 1 [file Table_1.docx]

| ***Supplementary File 1: Effects of multiple variables on clinical outcomes in univariate analysis*** | | |
| --- | --- | --- |
|  | ***Univariate analysis OR***(95% *CI*) | ***P value*** |
| **Age (years)** | 1.058（1.042-1.075） | <0.001 |
| **Sex** | 1.368（1.019-1.837） | 0.037 |
| **NYHA grading** | 3.549（2.675-4.709） | <0.001 |
| **Heart rate on admission, bpm** | 1.002（0.996-1.008） | 0.552 |
| **SBP on admission, mmHg** | 0.990（0.984-0.997） | 0.004 |
| **TYG,** **per 1 score increase** | 1.266（1.031-1.554） | 0.024 |
| **TYG,** **as a categories variable** |  |  |
| **Tertile1** | Reference | - |
| **Tertile2** | 1.054（0.726-1.531） | 0.781 |
| **Tertile3** | 1.301（0.911-1.860） | 0.148 |
| **GWTG-HF score** | 1.099（1.078-1.121） | <0.001 |
| **Albumin, g/L** | 0.866（0.839-0.894） | <0.001 |
| **TBIL, umol/L** | 1.008（0.997-1.020） | 0.145 |
| **LDL, mmol/L** | 0.941（0.806-1.098） | 0.437 |
| **BUN, mg/dL** | 1.007（1.006-1.008） | <0.001 |
| **Creatinine, mg/dL** | 1.511（1.374-1.661） | <0.001 |
| **Uric Acid, umol/L** | 1.004（1.003-1.005） | <0.001 |
| **Haemoglobin, g/L** | 0.976（0.970-0.981） | <0.001 |
| **Serum sodium, mmol/L** | 0.906（0.879-0.934） | <0.001 |
| **cTNI, ng/ml** | 1.032（1.021-1.042） | <0.001 |
| **NT-proBNP, per 100pg/ml** | 1.008（1.006-1.010） | <0.001 |
| **LVEF, %** | 0.989（0.975-1.002） | 0.105 |
| **CAD** | 1.524（1.068-2.175） | 0.020 |
| **Hypertension** | 0.814（0.605-1.096） | 0.175 |
| **AF** | 0.648（0.458-0.917） | 0.014 |
| **DM** | 1.282（0.947-1.736） | 0.108 |
| **COPD** | 0.781（0.541-1.127） | 0.186 |
| **Smoking** | 0.589（0.406-0.856） | 0.006 |
| **ACE-I/ARB/ARNI** | 0.333（0.246-0.451） | <0.001 |
| **Beta blockers** | 1.308（0.869-1.970） | 0.198 |
| **Diuretic** | 0.548（0.389-0.773） | 0.001 |
| **Aldosterone antagonists** | 0.673（0.473-0.958） | 0.028 |

Abbreviations: ACE-I, angiotensin-converting enzyme inhibitors; AF, atrial fibrillation; ARB, angiotensin II receptor blockers; ARNI, angiotensin receptor blocker-neprilysin inhibitors; BUN, blood urea nitrogen; CAD, coronary artery disease; COPD, chronic obstructive pulmonary disease; cTNI, cardiac troponin I; eGFR, estimated glomerular filtration rate; FPG, fasting plasma glucose; GWTG-HF, Get With the Guidelines-Heart Failure; HbA1c, glycated hemoglobin; LDL, low-density lipoprotein; LVEF, left ventricular ejection fraction; NT-proBNP, N‐terminal brain natriuretic peptide; SBP, systolic blood pressure; T2DM, type 2 diabetes mellitus; TBIL, total bilirubin; TyG, triglyceride-glucose.
