# Supplementary material for: Triglyceride glucose index and its combination with the Get with the Guidelines-Heart Failure score in predicting the prognosis in patients with heart failure
Source: Front Nutr. 2022 Sep 8;9:950338. doi: 10.3389/fnut.2022.950338 (PMC9493032; doi:10.3389/fnut.2022.950338)
Supplement: Supplementary file 2 [file Table_2.DOCX]

| ***Supplementary File 2: Effects of multiple variables on clinical outcomes in multivariate analysis*** | | |
| --- | --- | --- |
|  | ***multivariate analysis OR***(95% *CI*) | ***P value*** |
| **Age, years** | 1.058（1.039-1.077） | <0.001 |
| **Sex** | 0.935（0.650-1.346） | 0.719 |
| **NYHA grading** | 2.317（1.686-3.183） | <0.001 |
| **Heart rate on admission, bpm** | 0.998（0.989-1.007） | 0.656 |
| **SBP on admission, mmHg** | 1.000（0.991-1.009） | 0.959 |
| **TYG index,** **per 1 score increase** | 1.886（1.421-2.501） | <0.001 |
| **Albumin, g/L** | 0.923（0.885-0.963） | <0.001 |
| **TBIL, umol/L** | 1.017（1.002-1.031） | 0.024 |
| **LDL, mmol/L** | 1.022（0.861-1.212） | 0.807 |
| **BUN, mg/dL** | 1.004（1.003-1.006） | <0.001 |
| **Creatinine, mg/dL** | 0.924（0.735-1.163） | 0.502 |
| **Uric Acid, umol/L** | 1.001（1.000-1.002） | 0.034 |
| **Haemoglobin, g/L** | 0.992（0.984-1.000） | 0.053 |
| **Serum sodium, mmol/L** | 0.966（0.931-1.002） | 0.061 |
| **cTNI, ng/ml** | 1.025（1.012-1.038） | <0.001 |
| **NT-proBNP, per 100pg/ml** | 1.002（1.000-1.004） | 0.049 |
| **LVEF, %** | 0.962（0.933-0.992） | 0.013 |
| **CAD** | 0.975（0.635-1.497） | 0.909 |
| **Hypertension** | 0.643（0.443-0.932） | 0.020 |
| **AF** | 0.962（0.647-1.432） | 0.849 |
| **DM** | 0.805（0.540-1.199） | 0.286 |
| **COPD** | 0.924（0.616-1.387） | 0.704 |
| **Smoking** | 0.716（0.467-1.096） | 0.124 |
| **ACE-I/ARB/ARNI** | 0.868（0.526-1.433） | 0.580 |
| **Beta blockers** | 1.399（0.798-2.454） | 0.241 |
| **Diuretic** | 0.669（0.442-1.012） | 0.057 |
| **Aldosterone antagonists** | 0.339（0.177-0.650） | 0.001 |

Abbreviations: ACE-I, angiotensin-converting enzyme inhibitors; AF, atrial fibrillation; ARB, angiotensin II receptor blockers; ARNI, angiotensin receptor blocker-neprilysin inhibitors; BUN, blood urea nitrogen; CAD, coronary artery disease; COPD, chronic obstructive pulmonary disease; cTNI, cardiac troponin I; eGFR, estimated glomerular filtration rate; FPG, fasting plasma glucose; GWTG-HF, Get With the Guidelines-Heart Failure; HbA1c, glycated hemoglobin; LDL, low-density lipoprotein; LVEF, left ventricular ejection fraction; NT-proBNP, N‐terminal brain natriuretic peptide; SBP, systolic blood pressure; T2DM, type 2 diabetes mellitus; TBIL, total bilirubin; TyG, triglyceride-glucose.
