# Supplementary material for: Triglyceride glucose index and its combination with the Get with the Guidelines-Heart Failure score in predicting the prognosis in patients with heart failure
Source: Front Nutr. 2022 Sep 8;9:950338. doi: 10.3389/fnut.2022.950338 (PMC9493032; doi:10.3389/fnut.2022.950338)
Supplement: Supplementary file 3 [file Table_3.DOCX]

| ***Supplementary File 3: Effects of multiple variables on clinical outcomes in multivariate analysis*** | | |
| --- | --- | --- |
|  | ***multivariate analysis OR***(95% *CI*) | ***P value*** |
| **Age, years** | 1.055（1.036-1.075） | <0.001 |
| **Sex** | 0.977（0.681-1.401） | 0.900 |
| **NYHA grading** | 2.287（1.665-3.140） | <0.001 |
| **Heart rate on admission, bpm** | 0.998（0.989-1.008） | 0.732 |
| **SBP on admission, mmHg** | 1.000（0.991-1.009） | 0.980 |
| **TYG,** **as a categories variable** |  |  |
| **Tertile1** | Reference | - |
| **Tertile2** | 1.536（1.002-2.354） | 0.049 |
| **Tertile3** | 2.076（1.284-3.354） | 0.003 |
| **Albumin, g/L** | 0.925（0.887-0.964） | <0.001 |
| **TBIL, umol/L** | 1.015（1.000-1.029） | 0.050 |
| **LDL, mmol/L** | 1.058（0.891-1.255） | 0.522 |
| **BUN, mg/dL** | 1.004（1.002-1.006） | <0.001 |
| **Creatinine, mg/dL** | 0.929（0.740-1.166） | 0.525 |
| **Uric Acid, umol/L** | 1.001（1.000-1.002） | 0.028 |
| **Haemoglobin, g/L** | 0.992（0.985-1.000） | 0.066 |
| **Serum sodium, mmol/L** | 0.962（0.928-0.998） | 0.037 |
| **cTNI, ng/ml** | 1.027（1.014-1.040） | <0.001 |
| **NT-proBNP, per 100pg/ml** | 1.002（1.000-1.004） | 0.043 |
| **LVEF, %** | 0.964（0.935-0.994） | 0.019 |
| **CAD** | 0.985（0.642-1.510） | 0.944 |
| **Hypertension** | 0.644（0.445-0.934） | 0.020 |
| **AF** | 0.938（0.632-1.393） | 0.752 |
| **DM** | 0.945（0.645-1.386） | 0.773 |
| **COPD** | 0.901（0.601-1.350） | 0.612 |
| **Smoking** | 0.718（0.469-1.098） | 0.126 |
| **ACE-I/ARB/ARNI** | 0.888（0.538-1.464） | 0.640 |
| **Beta blockers** | 1.394（0.797-2.440） | 0.245 |
| **Diuretic** | 0.671（0.443-1.017） | 0.060 |
| **Aldosterone antagonists** | 0.346（0.181-0.662） | 0.001 |

Abbreviations: ACE-I, angiotensin-converting enzyme inhibitors; AF, atrial fibrillation; ARB, angiotensin II receptor blockers; ARNI, angiotensin receptor blocker-neprilysin inhibitors; BUN, blood urea nitrogen; CAD, coronary artery disease; COPD, chronic obstructive pulmonary disease; cTNI, cardiac troponin I; eGFR, estimated glomerular filtration rate; FPG, fasting plasma glucose; GWTG-HF, Get With the Guidelines-Heart Failure; HbA1c, glycated hemoglobin; LDL, low-density lipoprotein; LVEF, left ventricular ejection fraction; NT-proBNP, N‐terminal brain natriuretic peptide; SBP, systolic blood pressure; T2DM, type 2 diabetes mellitus; TBIL, total bilirubin; TyG, triglyceride-glucose.
